# Motivational–addictive profiles of nonsuicidal self-injury in Chinese youth: a cluster analysis with validation using external correlates

**DOI:** 10.3389/fpsyt.2026.1785425

**Published:** 2026-04-30

**Authors:** Mengying Wu, Jian Lin, Jialing Huang, Haisheng Zhang, Jian Xie, Chenyi Yan, Shu Yu, Changchun Hu

**Affiliations:** 1 School of the Fourth Clinical Medicine, Zhejiang Chinese Medical University, Hangzhou, China; 2 Department of Clinical Psychology, Affiliated Hangzhou First People’s Hospital, Westlake University School of Medicine, Hangzhou, China; 3 Shaoxing Seventh People’s Hospital, Affiliated Mental Health Center, Medical College of Shaoxing University, Shaoxing, China

**Keywords:** adolescents, behavioral addiction, cluster analysis, emotion regulation, nonsuicidal self-injury

## Abstract

**Background:**

Nonsuicidal self-injury (NSSI) is heterogeneous, yet clinically useful exploratory subgrouping remains limited. We examined whether motivational and addiction-like features could delineate meaningful data-driven NSSI profiles and relate to clinically relevant outcomes.

**Methods:**

We clustered six motive dimensions of the Ottawa Self-Injury Inventory (OSI; F1–F6) and an addiction-like score in adolescents (N = 311; age range: 12–21 years; mean age = 15.15 years, SD = 2.60). A three-cluster solution was retained based on a multi-criterion evaluation of internal fit indices, cluster-size balance, and clinical interpretability, with the WCSS elbow plot showing a clear inflection at k = 3. Outcomes were tested in age/sex-adjusted models (ordinal frequency and pain via proportional-odds; Patient Health Questionnaire-9[PHQ-9]/Generalized Anxiety Disorder 7[GAD-7] scores were standardized in the full sample [n = 311]).

**Results:**

Three data-driven profiles emerged: Emotion-regulation (n = 112), Multi-motivated/high-addiction (n = 79), and Lower-severity (n = 120). Versus Lower-severity, both higher-severity clusters showed higher monthly (OR = 3.58 and 2.38) and yearly NSSI frequency (OR = 2.91 and 1.96). Pain perception was lower in the high-addiction cluster (OR = 0.49). PHQ-9 and GAD-7 were higher in the high-addiction cluster, and GAD-7 was also higher in the emotion-regulation cluster.

**Conclusions:**

Motivational–addictive features identified interpretable, data-driven NSSI profiles associated with frequency, pain, and affective symptoms; these exploratory profiles require replication, longitudinal follow-up, and independent validation before further clinical inferences can be drawn.

## 1 Introduction

Nonsuicidal self-injury (NSSI) is defined as the deliberate destruction of one’s own body tissue with the intention of coping rather than ending life, with common forms including cutting, hitting, scratching, or burning (1). It occurs across a range of psychiatric disorders, particularly mood and personality disorders, but can also present in individuals without formal diagnoses (2). NSSI has rapidly increased in prevalence among adolescents and is listed in the DSM-5 as a condition warranting further investigation, attracting substantial attention in global mental health research (3). Epidemiological studies suggest a lifetime prevalence of 17%–18% in non-clinical adolescent populations (4, 5), with rates exceeding 60% in clinical populations, where repetitive behaviors are especially common (6). In China, a meta-analysis estimated the prevalence of NSSI among middle and high school students at 22.4% (7), and more recent surveys report rates exceeding 50% in some urban clinical samples (8, 9), far exceeding the international average and posing a significant public health concern (10). Recent research supports that NSSI is a heterogeneous behavior that varies in functions, severity, and associated clinical features. Emotion regulation is consistently identified as a core process in NSSI, although functional profiles differ meaningfully across individuals. Recent subgrouping studies further suggest that empirically derived NSSI profiles may be clinically informative, highlighting the need for more integrative classification approaches (11–13).

The psychological mechanisms underlying NSSI are highly complex, encompassing a wide array of motivations such as emotion regulation, self-punishment, sensory stimulation, and interpersonal influence. Some individuals exhibit compulsive or impulsive traits in their NSSI behaviors, suggesting addiction-like features. Notably, motivational dimensions and addiction-like features represent two complementary yet conceptually distinct constructs: the former characterizes the intentional functional purpose of engaging in NSSI (i.e., why an individual performs the behavior), while the latter captures reinforcement- and compulsivity-related maintenance patterns (i.e., the extent to which the behavior becomes repetitive, habitual, or difficult to control). This addictive-like construct is operationally independent of the motivational dimensions of NSSI, with both assessed as discrete subscales in the OSI. In this study, terms such as “addiction-like features,” “addictive profiles,” and “addiction severity” are used descriptively to denote the addictive features dimension assessed by the Ottawa Self-Injury Inventory (OSI), namely compulsive, reinforcement-related, or habit-like characteristics of NSSI, and do not imply that NSSI is formally classified as an addictive disorder (14). However, prior research has often treated NSSI as a relatively homogeneous phenomenon and has insufficiently integrated motivational and addiction-like characteristics. Such limitations may hinder the identification of clinically meaningful subgroups and the development of precision-targeted interventions. Recent data-driven approaches, including clustering and latent class analyses, offer a useful strategy for addressing this heterogeneity (13, 15). We further selected depression and pain perception as external correlates because both are clinically and theoretically relevant correlates of NSSI. Depressive symptoms are strongly linked to NSSI severity and clinical burden in adolescents with mood disorders, whereas altered pain perception has been repeatedly implicated in the maintenance and neurobehavioral mechanisms of NSSI (16–18). Therefore, examining these variables may help determine whether the identified data-driven profiles differ not only in motivational and addiction-like features, but also in clinically meaningful external characteristics.

Motivation and addiction-like characteristics were selected because they capture two complementary dimensions of NSSI heterogeneity: the functional purpose of the behavior and its reinforcement-/compulsivity-related maintenance pattern. Recent subgrouping research suggests that empirically derived profiles of NSSI may be clinically informative, while psychometric work using the Ottawa Self-Injury Inventory (OSI) supports the distinction between functional and addictive-feature dimensions. Therefore, integrating both domains may provide a more clinically informative basis for subgroup identification than considering either domain alone (11, 14, 19).

To address these gaps, the current study aimed to (1): use cluster analysis based on motivational dimensions (F1–F6) and addiction-like traits to identify data-driven behavioral profiles; and (2) examine differences in NSSI frequency, pain perception, and emotional symptoms across data-driven profiles. This data-driven analysis in a Chinese sample expands cross-cultural evidence on NSSI typologies and may inform subtype-focused assessment and future intervention research.

## 2 Methods

### 2.1 Participants

This cross-sectional study was conducted between August 2019 and December 2022 at the Department of Clinical Psychology, Affiliated Hangzhou First People’s Hospital, Westlake University School of Medicine, a tertiary general hospital with a specialized clinical psychology service in Hangzhou, eastern China. Participants were consecutively recruited during routine clinical intake from both outpatient and inpatient clinical services of the department, which provides comprehensive mental health assessment and treatment for adolescents and young adults with mood and other psychiatric disorders. A total of 311 treatment-naïve adolescents and young adults aged 12–21 years were enrolled, all of whom were assessed during their first clinical visit and had no prior history of psychotropic medication use at the time of study assessment. The mean age of the sample was 15.15 years (SD = 2.60), with an age range of 12–21 years, and the sample was predominantly female (80.7%).

Inclusion criteria were strictly defined as follows: (1) aged 12 to 21 years at the time of assessment; (2) a primary diagnosis of a mood disorder (i.e., major depressive disorder [MDD] or bipolar depression [BD]) confirmed according to the Diagnostic and Statistical Manual of Mental Disorders (5th edition, DSM-5) criteria; (3) a history of at least one nonsuicidal self-injury (NSSI) episode within the 12 months prior to recruitment; (4) sufficient cognitive capacity to complete all study assessments independently; and (5) provision of written informed consent by the participant and their legal guardian (for minors aged <18 years).

Exclusion criteria were: (1) a current or past primary diagnosis of a schizophrenia spectrum or other psychotic disorder; (2) severe cognitive impairment (e.g., intellectual disability, traumatic brain injury with persistent cognitive deficits) that impairs assessment completion; (3) a primary diagnosis of autism spectrum disorder; (4) a history of suicidal attempt with medical hospitalization within the 3 months prior to recruitment; and (5) current use of psychotropic medications or participation in other psychological intervention studies at the time of assessment.

*A priori* sample size estimation using G*Power 3.1 indicated that a minimum of 270 participants would be required to detect a small-to-moderate effect size (f = 0.20) with 80% power at α = 0.05 in a three-group ANOVA. The final sample exceeded this threshold, ensuring adequate statistical power.

All study diagnoses were independently confirmed by two board-certified, experienced psychiatrists with specialized training in child and adolescent psychiatry, using the Chinese version of the Mini International Neuropsychiatric Interview (MINI) for structured diagnostic assessment. Discrepancies in diagnostic judgment were resolved via a consensus meeting with a third senior psychiatrist. Ethical approval was obtained from the institutional review board of Hangzhou First People’s Hospital (IRB No. 2020-K008-01), and the study was conducted in strict adherence to the ethical principles of the Declaration of Helsinki. Written informed consent was obtained from all participants and their legal guardians (for participants <18 years of age) prior to any study procedures.

### 2.2 Measures

A battery of standardized instruments was administered to assess sociodemographic background, psychiatric symptoms, NSSI behaviors, motivations, and addiction-like features.

#### 2.2.1 Demographic and clinical questionnaire

Gathered information on age, gender, education level, and family history of psychiatric disorders.

#### 2.2.2 Mini international neuropsychiatric interview (Chinese version)

Used for mood disorder diagnosis and to rule out major psychiatric comorbidities. The Chinese MINI show’s strong inter-rater reliability (κ > 0.80) in adolescent psychiatric populations (20).

#### 2.2.3 Ottawa self-injury inventory

Assessed NSSI, pain perception, six motivational dimensions (F1–F6), and a standalone addiction-like feature dimension that is operationally distinct from the motivational subscales (14, 19). The addiction-like feature dimension was measured via 7 items rated on a 0–4 Likert scale (total score range: 0–28), quantifying compulsive engagement, difficulty controlling NSSI behavior, repetitive performance, and reinforcement of NSSI via relief from negative affect or physical sensation; higher scores indicate stronger addiction-like characteristics. F1 Internal Emotion Regulation: items 4, 10, 19, 21, 25 (alleviating negative affect/tension via NSSI); F2 Social Influence: items 3, 5, 9, 11, 12, 14, 15, 20, 27 (interpersonal communication/influence); F3 External Emotion Regulation: items 1, 8, 13, 17, 26 (changing internal state via physical sensation; e.g., stopping numbness/interrupting distress); F4 Sensation Seeking: items 2, 7, 29 (seeking stimulation/arousal); F5 Anti-suicide: items 23, 24 (resisting suicidal urges/preventing attempts); F6 Self-punishment: item 6 (self-directed punishment/blame) (14, 19, 21). Pain perception during NSSI was assessed using the OSI single item: “Do you feel physical pain when you harm yourself?” Responses were rated on a 0–4 ordinal scale (0 = Never, 1 = Rarely, 2 = Sometimes, 3 = Often, 4 = Always), with higher scores indicating greater perceived pain intensity. Pain perception was treated as an ordinal variable in primary analyses. In the present sample, internal consistency was high for the addiction-like feature subscale (α≈ 0.88; total score range: 0-28) and acceptable-to-excellent for motivational subscales (α≈ 0.76-0.91). Here, addiction-related terminology refers specifically to the OSI addictive features dimension, a descriptive and measurement-based construct independent of the motivational dimensions, and is used without implying a formal addictive disorder diagnosis (14). Monthly NSSI frequency level was recorded as a 4-level ordinal variable (0–3; 0 = none in the past month; 1 = 1–4; 2 = 5–9; 3 = ≥10). Yearly NSSI frequency level was recorded as a 4-level ordinal variable (1–4; 1 = 1–4; 2 = 5–9; 3 = 10–20; 4 = >20). Monthly and yearly NSSI frequencies were originally collected as counts but recoded to ordinal categories to reduce skewness (15).

#### 2.2.4 Patient health questionnaire-9 and generalized anxiety disorder 7

Both Chinese versions demonstrated good internal consistency in this sample (α ≈ 0.88 and 0.91, respectively) (22–24).

### 2.3 Data analysis

All analyses were conducted in Python 3.8 and R 4.2.2. Participants with >10% missingness on core variables were excluded; overall missingness across study variables was <3%. Continuous variables are reported as mean ± SD or median [IQR], and categorical/ordinal variables as n (%).

**Figure 1:**
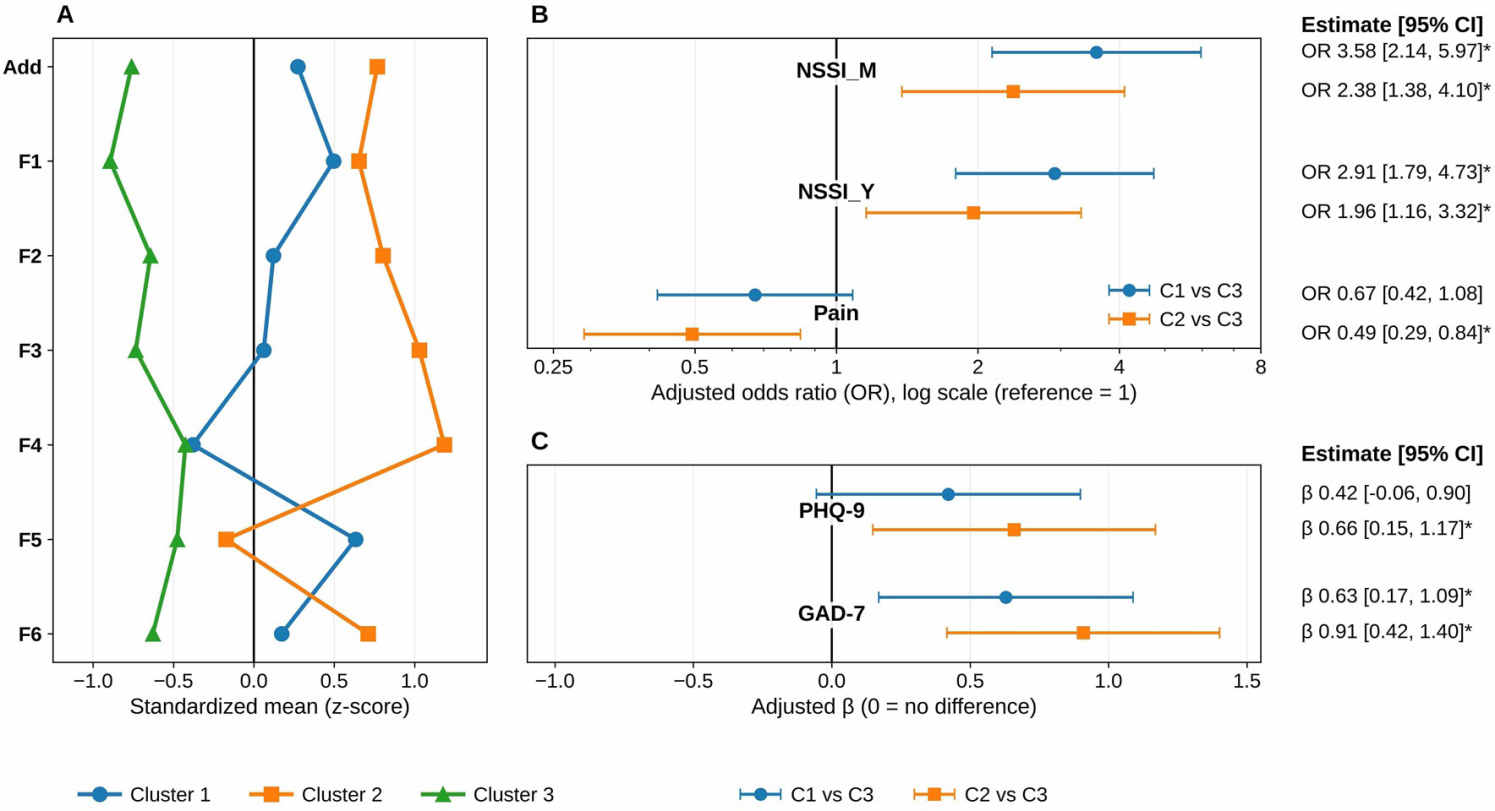
Standardized motivational–addictive profiles and group comparisons on NSSI frequency, pain perception, and affective symptoms across clusters.

Monthly and yearly NSSI frequency levels and pain perception were treated as ordinal outcomes and analyzed using cumulative logit (proportional-odds) models, adjusting for age and sex. Cluster membership was dummy-coded with Cluster 3 as the reference to estimate the primary contrasts (Cluster 1 vs Cluster 3; Cluster 2 vs Cluster 3) reported in **Table 1**, **Figure 1**. For distribution-robust sensitivity, group differences were additionally evaluated using Brunner-Munzel tests (two-group) and Kruskal-Wallis tests with Dunn-BH *post hoc* comparisons (multi-group); where parametric contrasts were retained for comparability, Welch’s t tests/Welch’s ANOVA with diagnostic checks (Shapiro–Wilk; Levene) and robust standard errors were used.

**Table 1:** Cluster associations with clinical and behavioral outcomes.

Outcome	Reference cluster	Comparison cluster	Effect (OR/β; 95% CI)	*p*-value	*q*-value
Monthly NSSI frequency	Cluster 3	Cluster 1	3.58 (2.14, 5.97)	0.001	0.003
Monthly NSSI frequency	Cluster 3	Cluster 2	2.38 (1.38, 4.10)	0.002	0.004
Yearly NSSI frequency	Cluster 3	Cluster 1	2.91 (1.79, 4.73)	0.001	0.003
Yearly NSSI frequency	Cluster 3	Cluster 2	1.96 (1.16, 3.32)	0.012	0.014
Pain perception level	Cluster 3	Cluster 1	0.67 (0.42, 1.08)	0.102	0.102
Pain perception level	Cluster 3	Cluster 2	0.49 (0.29, 0.84)	0.009	0.013
PHQ-9	Cluster 3	Cluster 1	0.42 (-0.06, 0.90)	0.087	0.087
PHQ-9	Cluster 3	Cluster 2	0.66 (0.15, 1.17)	0.013	0.017
GAD-7	Cluster 3	Cluster 1	0.63 (0.17, 1.09)	0.009	0.017
GAD-7	Cluster 3	Cluster 2	0.91 (0.42, 1.40)	0.001	0.004

Monthly and yearly NSSI frequency and pain perception were modeled as ordinal outcomes using proportional-odds models adjusted for age and sex; effects are odds ratios (OR) for higher outcome categories. PHQ-9 and GAD-7 were analyzed as standardized continuous outcomes; effects are β (mean difference in SD units). Cluster 3 is the reference group. q-values are Benjamini–Hochberg FDR-adjusted within panel (Panel B: 6 tests; Panel C: 4 tests).

Multiple testing was controlled using Benjamini–Hochberg FDR (*q* = 0.05) within predefined families: OSI motives (F1–F6) were FDR-corrected within-domain, and external criteria in **Figure 1** were FDR-corrected within panel (Panel B: 6 tests across NSSI_M, NSSI_Y, and Pain; Panel C: 4 tests across PHQ-9 and GAD-7). Effect sizes are reported with 95% confidence intervals (Hedges’ g; ϵ²; ORs for ordinal models).

Clustering was performed using k-means on z-scored OSI motives (F1–F6) and addiction-like features, with the retained k = 3 solution selected using a multi-criterion approach that considered internal fit indices, cluster-size balance, and clinical interpretability (see Section 3.3 and **Supplementary Table 1**).

We tested age moderation using Cluster × Age models (Outcome ~ Cluster + Age + Cluster × Age + Sex) for pain, monthly/yearly NSSI frequency, and age of first NSSI. We also conducted stratified sensitivity analyses (12–17 vs 18–21 years) adjusting for sex (and age within stratum). Details are reported in the Supplement (**Supplementary Table 3**).

## 3 Results

In the present study, the identified clusters were evaluated using external correlates (i.e., variables not used in the clustering procedure), including NSSI frequency, pain perception, and affective symptoms. Because these analyses were conducted within the same sample rather than in an independent dataset, we refer to them as evidence of construct validity using external correlates, rather than external validation in the strict methodological sense.

### 3.1 Sample characteristics

The analytic sample comprised three data-driven clusters (N = 311; Cluster 1 n = 112, Cluster 2 n = 79, Cluster 3 n = 120; **Table 2**). Age distributions were comparable across clusters (median [IQR]: Cluster 1 = 15.0 [13.0–16.25], Cluster 2 = 14.0 [13.0–16.0], Cluster 3 = 14.0 [14.0–16.0] years; Kruskal–Wallis *p* = 0.73). The sample showed a female predominance (80.7%), which is commonly observed in clinically ascertained NSSI research. Meta-analytic evidence indicates higher NSSI prevalence among female adolescents in clinical samples, suggesting that our sex distribution may partly reflect clinical referral/help-seeking patterns (25, 26). Notably, the diagnostic composition was unevenly distributed across the three identified clusters, with Cluster 1 characterized by a predominance of MDD (59.8%) and Cluster 2 by a predominance of BD (83.5%), while Cluster 3 had an equal distribution of MDD and BD (50% each) (**Table 2**). Diagnostic composition varied across clusters (**Table 2**) and is considered as a potential confound in interpretation. Monthly NSSI frequency level is ordinal (0–3; 0 = none in the past month; 1 = 1–4; 2 = 5–9; 3 = ≥10). Yearly NSSI frequency level is ordinal (1–4; 1 = 1–4; 2 = 5–9; 3 = 10–20; 4 = >20). Monthly and yearly NSSI frequencies were recoded to ordinal categories to reduce skewness.

**Table 2:** Sample characteristics by cluster.

Characteristic	Cluster 1	Cluster 2	Cluster 3	Total
Sample size, n	112	79	120	311
Sex (M/F), n (%)	15 (13.4%)/97 (86.6%)	16 (20.3%)/63 (79.7%)	29 (24.2%)/91 (75.8%)	60 (19.3%)/251 (80.7%)
Age, median [IQR]	15 [13.0, 16.2]	14 [13.0, 16.0]	14 [14.0, 16.0]	15 [13.0, 16.0]
Diagnosis, n (%)				
MDD	67 (59.8%)	13 (16.5%)	60 (50%)	140 (44.9%)
BD	45 (40.2%)	66 (83.5%)	60 (50%)	171 (55.1%)
Monthly NSSI level, n (%)				
Level 0	8 (7.1%)	8 (10.1%)	25 (20.8%)	41 (13.2%)
Level 1	50 (44.6%)	37 (46.8%)	68 (56.7%)	155 (49.8%)
Level 2	26 (23.2%)	24 (30.4%)	21 (17.5%)	71 (22.8%)
Level 3	28 (25.0%)	10 (12.7%)	6 (5.0%)	44 (14.1%)
Yearly NSSI level, n (%)				
Level 1	17 (15.2%)	18 (22.8%)	40 (33.3%)	75 (24.1%)
Level 2	26 (23.2%)	17 (21.5%)	38 (31.7%)	81 (26.0%)
Level 3	48 (42.9%)	35 (44.3%)	34 (28.3%)	117 (37.6%)
Level 4	21 (18.8%)	9 (11.4%)	8 (6.7%)	38 (12.2%)
Pain level, n (%)				
Level 0	21 (18.8%)	17 (21.5%)	15 (12.5%)	53 (17.0%)
Level 1	50 (44.6%)	40 (50.6%)	51 (42.5%)	141 (45.3%)
Level 2	27 (24.1%)	15 (19.0%)	29 (24.2%)	71 (22.8%)
Level 3	7 (6.2%)	3 (3.8%)	16 (13.3%)	26 (8.4%)
Level 4	7 (6.2%)	4 (5.1%)	9 (7.5%)	20 (6.4%)

Monthly NSSI frequency level (0–3): 0 = none in the past month; 1 = 1–4; 2 = 5–9; 3 = ≥10. Yearly NSSI frequency level (1–4): 1 = 1–4; 2 = 5–9; 3 = 10–20; 4 = >20. Pain level (0–4): higher levels indicate greater pain. Values are n (%) unless otherwise indicated.

### 3.2 Correlation analysis

Spearman correlations (all *p* < 0.001 after correction) showed that monthly and yearly NSSI frequency were positively associated with addiction score (ρ= 0.42 and approximately 0.45, respectively) and internal emotion regulation (F1, ρ = 0.38 and approximately 0.32, respectively). Pain perception was negatively correlated with addiction score (ρ = −0.30) and social influence (F2, ρ = −0.29). These correlations are reported for descriptive context and were not used as primary criteria for cluster selection.

### 3.3 Cluster analysis of NSSI data-driven profiles and cluster profiles

**Figure 2:**
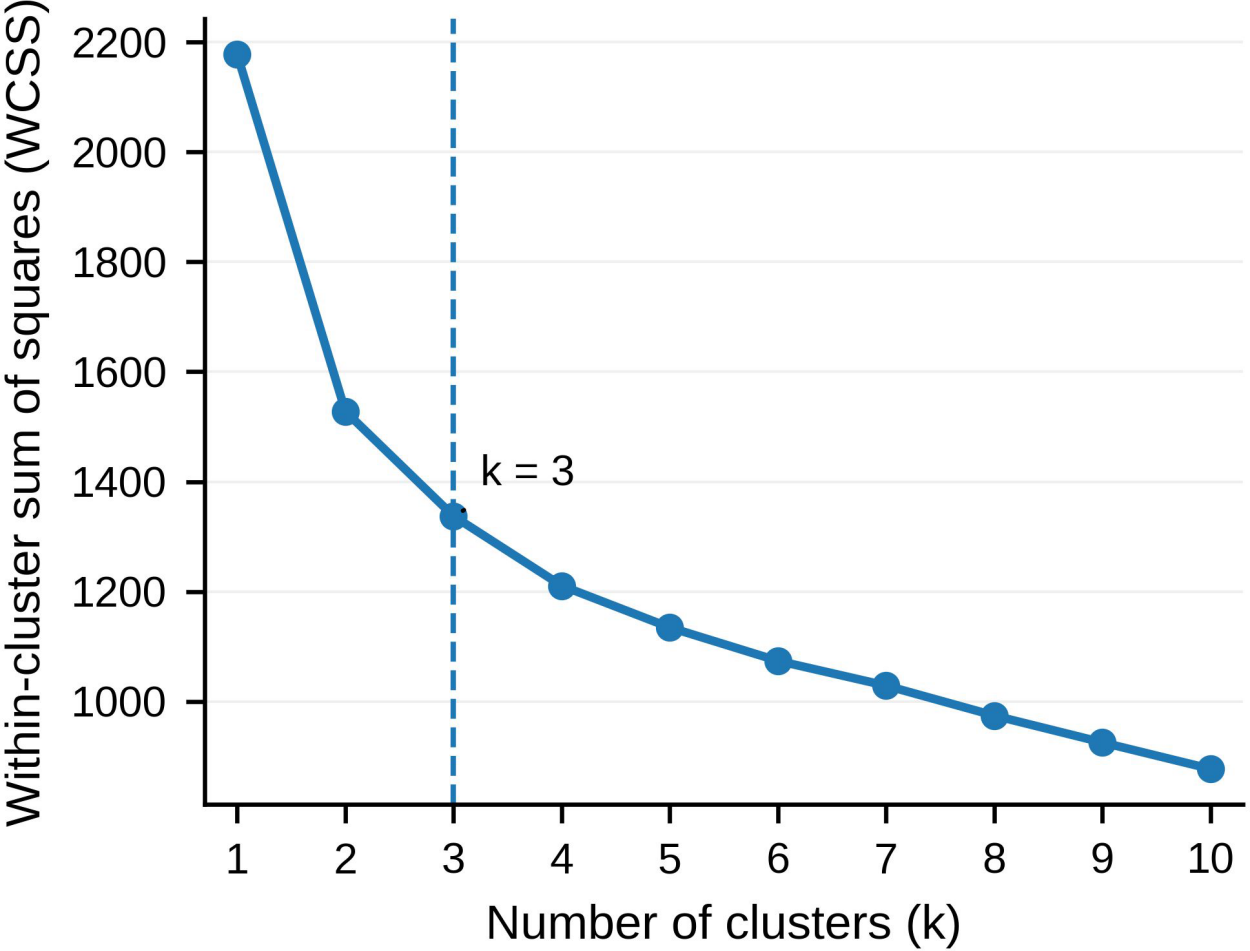
Within-cluster sum of squares (WCSS) plot for cluster number selection.

**Figure 3:**
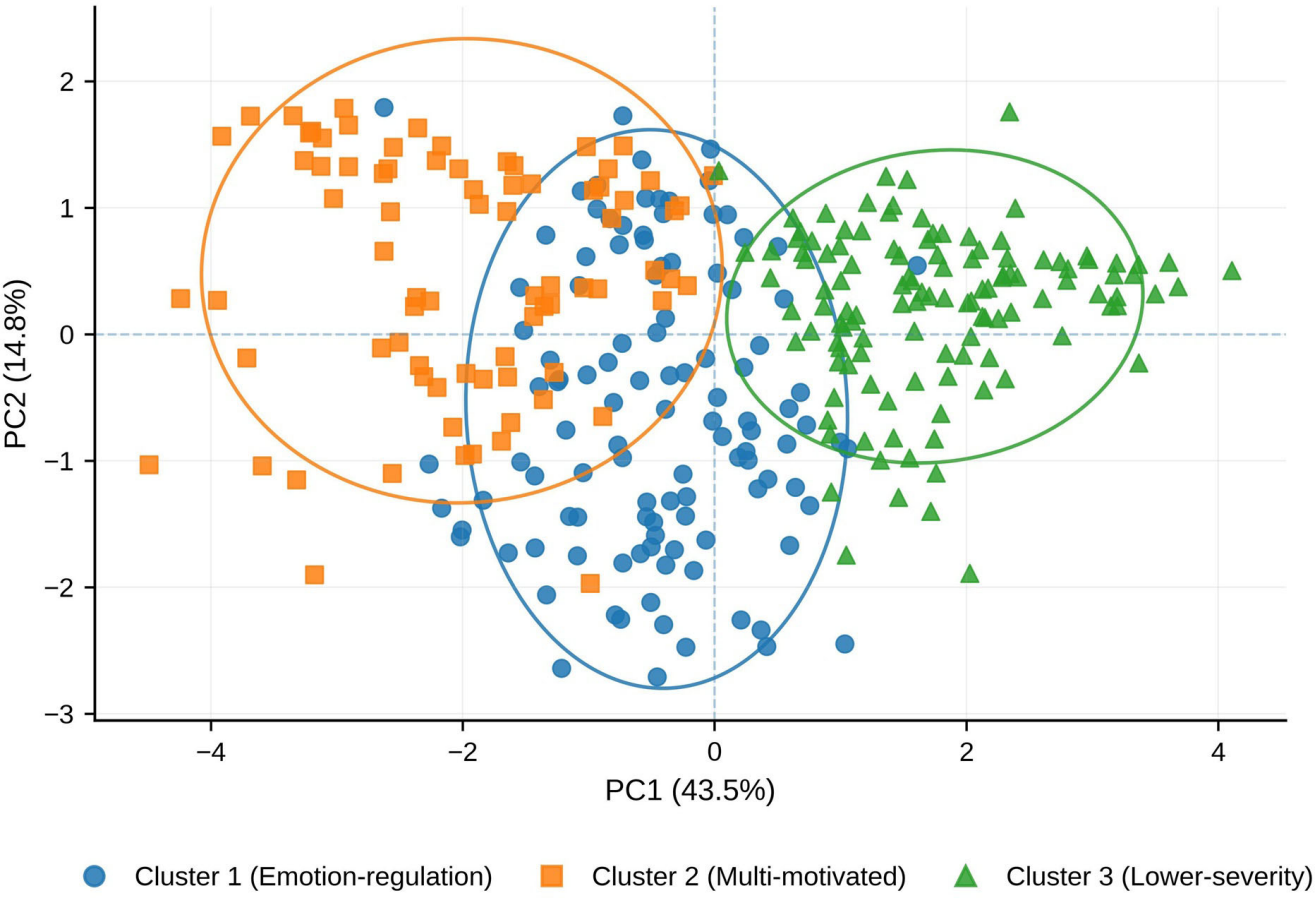
Two-dimensional principal component analysis (PCA) plot of the three identified NSSI clusters.

K-means clustering was performed on z-standardized OSI motivational subscales (F1–F6) and the addiction-like score to identify data-driven profiles of NSSI. The retained three-cluster solution was selected using a multi-criterion evaluation that considered internal fit indices, cluster-size balance, and clinical interpretability, rather than relying on any single index alone (**Supplementary Table 1**). Specifically, fit indices across k = 2–8 were compared using within-cluster sum of squares (WCSS), silhouette coefficient, Calinski-Harabasz index, Davies-Bouldin index, and the gap statistic. Although several internal indices favored a two-cluster solution, the three-cluster solution was retained because it provided acceptable cluster balance, clearer clinical interpretability, and more informative separation across external correlates. A clear inflection at k = 3 on the WCSS elbow plot served as the key visual support for the retained solution (**Figure 2**), and principal component analysis (PCA) further illustrated separation of the three clusters in reduced feature space (**Figure 3**). The three clusters were distinctly differentiated in motivational and addiction-like features (**Table 3**; **Figure 1A**): Cluster 1 (Emotion-regulation, n = 112) was dominated by internal emotion regulation motives (F1) with moderate addiction-like features; Cluster 2 (Multi-motivated/high-addiction, n = 79) showed globally elevated endorsement of all motives with the highest addiction severity; Cluster 3 (Lower-severity, n = 120) exhibited uniformly low endorsement of all motives and the lowest addiction-like features. These motivational-addictive profiles were associated with graded clinical and behavioral differences in age- and sex-adjusted outcome models (**Figure 1B**; **Table 1**).

**Table 3:** Cluster profiles (addiction and OSI motives) and omnibus tests.

Variable	Cluster 1	Cluster 2	Cluster 3	Statistic	*p-value*	*ϵ²*
addiction	15.5[10, 19.0]	19 [14.0, 24.0]	5[2.0, 10.0]	126.63	<0.001	0.405
F1	25[22.0, 28.3]	27 [23.0, 32.0]	15[11.0, 19.0]	161.23	<0.001	0.517
F2	5 [2.0, 8.0]	8 [5.5, 10.0]	2 [0.0, 4.0]	101.77	<0.001	0.324
F3	7 [4.0, 9.0]	12 [9.0, 14.0]	3 [1.0, 5.0]	145.88	<0.001	0.467
F4	1 [0.0, 4.0]	9 [6.0, 12.0]	1 [0.0, 3.0]	119.56	<0.001	0.382
F5	4 [1.8, 7.0]	1 [0.0, 3.5]	0 [0.0, 2.0]	68.26	<0.001	0.215
F6	3 [1.8, 4.0]	4 [3.0, 4.0]	1 [0.0, 2.0]	94.12	<0.001	0.299

Values are presented as median [interquartile range, IQR]. Omnibus between-cluster comparisons were performed using the Kruskal–Wallis test (df = 2); the Statistic column reports the H value only. *P* values are two-sided; *P* < 0.001 is reported as “<0.001”. Effect size is reported as ϵ² (epsilon-squared). Addiction score was calculated as the sum of 7 items rated 0–4 (range 0–28), with higher scores indicating stronger addiction-like features. F1–F6 denote OSI motive subscale scores; higher scores indicate stronger endorsement of each motive. Operational definitions of F1–F6 are provided in the Measures section. Abbreviations. OSI, Ottawa Self-Injury Inventory; IQR, interquartile range.

Within-cluster sum of squares (WCSS) from k-means clustering on z-scored OSI motive subscales (F1–F6) and the addiction-like score (N = 311). The curve shows an inflection at k = 3 (dashed line), supporting a three-cluster solution.

Two-dimensional principal component analysis (PCA) plot showing participants projected onto the first two principal components derived from z-scored OSI motivational subscales (F1–F6) and the addiction-like feature (N = 311). PC1 and PC2 explained 43.5% and 14.8% of the total variance, respectively. Points are colored and shape-coded by cluster membership (Cluster 1: emotion-regulation; Cluster 2: multi-motivated; Cluster 3: Lower-severity). Ellipses indicate the 95% confidence region for each cluster.

### 3.4 Clinical and behavioral differences across clusters

Age- and sex-adjusted cumulative logit models, supported by Kruskal-Wallis and Dunn-BH tests, revealed significant graded differences across clusters in clinical and behavioral outcomes (**Table 1**; **Figures 1B, C**). Relative to Cluster 3 (Lower-severity), both Cluster 1 and Cluster 2 showed significantly higher monthly and yearly NSSI frequency, whereas only Cluster 2 showed reduced pain perception. Cluster 2 also had higher PHQ-9 and GAD-7 scores, while Cluster 1 showed significantly higher GAD-7 scores only. Sensitivity analyses yielded broadly consistent results, including diagnosis-adjusted models and age-related analyses (**Supplementary Tables 2**, **S3**).

(A) Standardized cluster profiles (z-scores) for OSI addiction-like features (Add) and six OSI motivation domains (F1–F6). (B) Adjusted odds ratios (ORs) with 95% CIs from cumulative logit models for ordinal outcomes (NSSI_M, NSSI_Y, Pain), comparing Cluster 1 vs Cluster 3 and Cluster 2 vs Cluster 3 (reference = Cluster 3), adjusted for age and sex (OR = 1, no difference). (C) Adjusted standardized coefficients (*β*) with 95% CIs for PHQ-9 and GAD-7, adjusted for age and sex (*β* = 0, no difference). Two-sided tests; * indicates BH–FDR–corrected *q* < 0.05 within panel (Panel B: 6 tests; Panel C: 4 tests).

## 4 Discussion

This study identified three clinically interpretable, data-driven NSSI profiles in Chinese youth via k-means clustering of OSI motivational dimensions (F1–F6) and addiction-like features, with clear graded differences in NSSI frequency, pain perception and affective symptoms across clusters. Relative to the Lower-severity cluster (Cluster 3), both the Emotion-regulation (Cluster 1) and Multi-motivated/high-addiction (Cluster 2) clusters exhibited higher monthly and yearly NSSI frequency; Cluster 2 additionally showed reduced pain perception and elevated depressive and anxiety symptoms, while Cluster 1 only had significantly elevated anxiety symptoms. These findings suggest that motivational-addictive features may help capture clinically relevant heterogeneity in adolescent NSSI presentations, and provide cross-cultural support for an integrative profiling framework that jointly considers functional motives and addiction-like characteristics (11).

### 4.1 Comparison with existing literature

The Emotion-regulation cluster (Cluster 1) aligns closely with the dominant functional model of NSSI that has long been the cornerstone of self-injury research, which posits NSSI as a core emotion regulation strategy to downregulate acute negative affect and relieve aversive internal states (5, 27, 28). This profile echoes cross-cultural and clinical findings that identify internal emotion regulation as the most frequently endorsed motive for NSSI across adolescent populations (11, 13), further validating the centrality of this functional dimension in NSSI heterogeneity among Chinese youth. In contrast, the Multi-motivated/high-addiction cluster (Cluster 2) is consistent with prior accounts of compulsive, habit-like NSSI patterns characterized by broad motivational repertoires, diminished pain salience, and reinforcement-related maintenance processes (14, 29, 30)—a subtype that has been underrecognized in traditional function-based subtyping but highlighted as a key marker of NSSI severity in OSI scale validation research (14, 19). This cluster directly addresses a critical gap in existing work, which has insufficiently integrated addictive-like maintenance features with functional motives despite their operational independence (14). The Lower-severity cluster (Cluster 3) likely reflects milder, situationally driven NSSI presentations, consistent with evidence from latent profile analysis that exploratory NSSI in adolescents is heterogeneous and often transient (13, 15), and aligns with findings of low-motive NSSI in less severe clinical samples (6).

Notably, the present study does not seek to replace established function-based NSSI models; instead, it extends these frameworks by incorporating addiction-like characteristics as a distinct, complementary dimension of NSSI heterogeneity (11, 19). Prior subtyping research has primarily focused on functional motives to delineate NSSI profiles (11, 13), but this work has not fully accounted for the maintenance of NSSI behavior via reinforcement and compulsivity—constructs that capture how the behavior persists rather than why it is initiated (14). Our integrative motivational-addictive framework thus provides a more comprehensive characterization of NSSI heterogeneity, and our findings in a Chinese clinical sample further confirm the generalizability of this integrative approach beyond Western populations (10). Collectively, our results replicate and extend previous NSSI subtyping research by integrating motivational and addiction-like features to capture the full spectrum of NSSI functional heterogeneity in Chinese youth, responding to recent calls for more holistic classification approaches (11, 15).

### 4.2 Mechanistic implications

A clear motivational-addictive gradient was observed across clusters, consistent with a potential shift from affect-regulation-dominant NSSI—the core focus of traditional NSSI mechanistic research (27, 28)—to more pervasive, compulsive features in the high-addiction profile, a shift that has only recently been the subject of neurobiological investigation in NSSI research (29, 30). Reduced pain perception in Cluster 2 may reflect neurobiological mechanisms such as pain habituation, endogenous analgesia or altered salience learning (17, 18), but causal inferences are limited by the cross-sectional study design. Notably, the current clustering relied on self-reported motives and addiction-like features; whether these profiles map to distinct neurocognitive or neurobiological processes remains an unaddressed empirical question for future research. A critical unresolved issue is the temporal stability of these NSSI profiles: it is unknown whether individuals transition between clusters (e.g., from Emotion-regulation to Multi-motivated/high-addiction) over time, and what clinical or social factors drive such changes. Longitudinal designs tracking motives, reinforcement processes, pain responses and NSSI course are therefore needed to distinguish stable NSSI data-driven profiles from developmental stages.

### 4.3 Potential clinical relevance

From a hypothesis-generating perspective, the identified profiles may help refine phenotypic characterization of NSSI and structure future research on clinically relevant heterogeneity in youth presentations (11, 13, 15). In particular, integrating motivational and addiction-like features may provide a useful framework for examining why some adolescents present with more frequent NSSI, lower pain perception, or greater affective burden (14, 18, 19). However, these implications should be interpreted cautiously. The present study did not examine treatment response, prognostic outcomes, or longitudinal profile stability; therefore, any clinical utility of these exploratory, cluster-derived profiles remains unestablished and requires independent replication, longitudinal follow-up, and intervention-informed evaluation.

### 4.4 Limitations

Several limitations should be noted. First, the marked sex imbalance (80.7% female) may limit generalizability, particularly to male adolescents. This female predominance may also reflect specific clinical referral and help-seeking patterns in the urban Chinese setting of the present study, and the identified NSSI profiles may not be generalizable to male adolescents or to other clinical and community settings with different demographic compositions. Although sex was adjusted for in all primary models, we cannot exclude the possibility that subtype characterization and clustering structure may differ in more sex-balanced or male-enriched clinical cohorts. Future multi-site studies should replicate these profiles in samples with broader and more balanced sex composition and evaluate sex-stratified subtype stability and external validity. Second, self-reported measures and ordinal recoding of monthly/yearly NSSI frequency may introduce measurement error and reduce sensitivity. Notably, the key clustering variables (OSI motivational and addiction-like features) and all clinical validators (pain perception, PHQ-9/GAD-7 affective symptoms) were primarily based on self-report assessments, which may introduce recall bias, social desirability bias, or reporting bias—limitations that may be amplified in adolescent clinical populations with mood disorders. Third, although participants were first-visit and medication-naïve, unmeasured clinical factors and marked unevenness in diagnostic composition (MDD vs. BD) across clusters may still confound subtype–validator associations. Specifically, Cluster 2 had a disproportionately high proportion of BD cases (83.5%) relative to Cluster 1 (40.2% BD) and Cluster 3 (50% BD), which may influence the observed differences in NSSI characteristics and affective symptoms across clusters. To address this concern, we conducted diagnosis-adjusted sensitivity analyses (adjusting for age, sex, and diagnosis), and the overall pattern of findings remained broadly consistent (**Supplementary Table 2**), suggesting that the identified clusters are not reducible to diagnostic subtype alone; however, residual confounding cannot be excluded, and replication in diagnostically balanced samples is warranted. Fourth, the cross-sectional study design of the present research precludes definitive conclusions regarding the temporal stability of the identified NSSI profiles, developmental transitions between clusters over time, or the predictive validity of the motivational-addictive features for future NSSI severity or clinical outcomes. Causal inferences about the directional relationships between motivational-addictive features, NSSI behavior, pain perception, and affective symptoms are also not possible with this design. Although Cluster × Age tests did not support robust age moderation, the smaller 18–21 subgroup limited power for stratified inference (**Supplementary Table 3**) and longitudinal studies are needed to evaluate developmental stability and change in these data-driven profiles. Fifth, clustering may be sensitive to feature selection and algorithms; thus, external replication is needed. Notably, the current study did not conduct external validation in an independent sample. The validation of the identified exploratory NSSI profiles was restricted to construct and criterion-related validity using external correlates (e.g., NSSI frequency, pain perception, affective symptoms) within the same study sample, which represents a key methodological limitation of the present findings. Finally, the sample was recruited exclusively from a single tertiary clinical psychology service in urban Hangzhou, China, and the observed NSSI profiles may therefore reflect cultural norms, local mental health service access, and referral-context influences specific to this setting. Generalizability to non-clinical Chinese adolescents, rural populations, or adolescents from other cultural and socioeconomic contexts is thus limited. This further limits the ability to draw exploratory conclusions about NSSI subgrouping or clinical implications from the current cross-sectional data alone.

### 4.5 Future directions

Future research should address the study’s limitations and extend these findings through targeted methodological and analytical approaches. Studies should adopt longitudinal designs to test the temporal stability of these NSSI profiles, track inter-cluster transitions over time, and integrate richer phenotyping—including ecological momentary assessment, behavioral neurocognitive tasks, and objective markers of pain processing—to elucidate the underlying neurobiological mechanisms of distinct motivational-addictive NSSI profiles. Additionally, profile-tailored intervention studies are needed to evaluate whether personalized clinical approaches based on motivational and addiction-like features yield improved NSSI outcomes relative to standard care, which would validate the clinical utility of this subtyping framework. Further research should also perform broader and more diverse sampling across clinical settings and cultural contexts, with balanced sex and diagnostic composition, and explicitly measure social/digital exposures and key clinical covariates (e.g., comorbidity, treatment history) to clarify the generalizability of the identified NSSI profiles. Finally, rigorous external validation in independent clinical and community samples is required, alongside tests of alternative clustering algorithms and feature sets, to confirm the robustness of the three-cluster motivational-addictive structure of NSSI in youth.

## 5 Conclusion

These findings suggest that a motivational–addictive framework may help refine phenotypic characterization of NSSI in Chinese youth and generate hypotheses for future stratification research. However, the stability, prognostic value, and clinical utility of these cluster-derived profiles remain to be established through independent replication, longitudinal follow-up, and intervention-informed studies.

## Data Availability

The original contributions presented in the study are included in the article/**Supplementary Material**. Further inquiries can be directed to the corresponding author.
