# Supplementary material for: Motivational–addictive profiles of nonsuicidal self-injury in Chinese youth: a cluster analysis with validation using external correlates
Source: Front Psychiatry. 2026 Apr 30;17:1785425. doi: 10.3389/fpsyt.2026.1785425 (PMC13171530; doi:10.3389/fpsyt.2026.1785425)
Supplement: Supplementary file 1 [file Table1.docx]

**Supplementary Table S1. Fit indices across k (2-8)**

| **k** | **WCSS (inertia)** | **Silhouette** | **Calinski-Harabasz** | **Davies-Bouldin** | **Min cluster %** | **Max cluster %** | **Gap statistic** | **Gap SE** |
| --- | --- | --- | --- | --- | --- | --- | --- | --- |
| 2 | 1525.30 | 0.258 | 132.023 | 1.451 | 47.5884 | 52.4116 | 0.241 | 0.013 |
| 3 | 1315.33 | 0.209 | 100.885 | 1.760 | 25.7235 | 39.5498 | 0.252 | 0.030 |
| 4 | 1172.18 | 0.210 | 87.723 | 1.641 | 19.6141 | 35.3698 | 0.312 | 0.091 |
| 5 | 1080.38 | 0.165 | 77.649 | 1.674 | 16.3987 | 29.582 | 0.316 | 0.019 |
| 6 | 1004.89 | 0.169 | 71.151 | 1.619 | 15.1125 | 19.614 | 0.332 | 0.021 |
| 7 | 964.48 | 0.160 | 63.697 | 1.677 | 9.3248 | 19.614 | 0.320 | 0.022 |
| 8 | 909.14 | 0.163 | 60.364 | 1.576 | 9.0032 | 15.756 | 0.330 | 0.023 |

Notes. The three-cluster solution was retained based on a combined evaluation of internal fit indices, cluster-size balance, and clinical interpretability, as described in the main text (Section 3.3). WCSS denotes within-cluster sum of squares (K-means inertia; lower indicates better compactness). Silhouette and Calinski-Harabasz (CH) indices: higher values indicate better separation. Davies-Bouldin (DB) index: lower values indicate better separation/compactness. Gap statistic: higher values indicate greater separation relative to a null reference; values are shown with standard error (SE). Before clustering, all features (self-harm addiction and F1–F6) were z-standardized (mean = 0, SD = 1). K-means was fitted with n_init = 20, max_iter = 300, random_state = 42. The gap statistic was computed using the Tibshirani et al. approach with B = 20 reference datasets generated by uniform sampling within the feature-wise bounding box in the standardized space; reference K-means used n_init = 10, max_iter = 300, random_state = 42. Although several internal indices favored k = 2, the k = 3 solution was retained based on overall interpretability, clinically informative separation, and acceptable cluster sizes.

**Diagnosis-adjusted sensitivity analysis.**

Given the unequal diagnostic composition (MDD vs BD) across clusters, we conducted sensitivity analyses additionally adjusting for diagnosis. The main pattern of cluster-validator associations remained consistent (Supplementary Table S2). After age, sex, and diagnosis, Cluster 1 and Cluster 2 remained associated with higher monthly and yearly NSSI frequency relative to Cluster 3 (e.g., monthly frequency: OR = 3.75 and 6.37; yearly frequency: OR = 4.62 and 18.08). Cluster 2 continued to show lower pain perception than Cluster 3 (OR = 0.40), and affective symptoms remained elevated in Cluster 2 (PHQ-9 β = 0.79; GAD-7 β = 0.94); Cluster 1 also showed higher GAD-7 (β = 0.62).

**Supplementary Table S2. Diagnosis-adjusted associations between cluster membership and clinical validators (****adjusting for age, sex, and diagnosis).**

| **Outcome** | **Contrast (ref = Cluster 3)** | **Estimate** | **95% CI** | ***p*** |
| --- | --- | --- | --- | --- |
| Monthly NSSI frequency (ordinal) | Cluster 1 vs 3 | OR = 3.75 | 2.23-6.32 | <0.001 |
|  | Cluster 2 vs 3 | OR = 6.37 | 3.40-11.91 | <0.001 |
| Yearly NSSI frequency (ordinal) | Cluster 1 vs 3 | OR = 4.62 | 2.61-8.17 | <0.001 |
|  | Cluster 2 vs 3 | OR = 18.08 | 9.00-36.33 | <0.001 |
| Pain perception (ordinal) | Cluster 1 vs 3 | OR = 0.68 | 0.42-1.10 | 0.114 |
|  | Cluster 2 vs 3 | OR = 0.40 | 0.23-0.70 | 0.001 |
| PHQ-9 (z-score) | Cluster 1 vs 3 | β = 0.37 | -0.11-0.84 | 0.128 |
|  | Cluster 2 vs 3 | β = 0.79 | 0.26-1.31 | 0.003 |
| GAD-7 (z-score) | Cluster 1 vs 3 | β = 0.62 | 0.15-1.09 | 0.011 |
|  | Cluster 2 vs 3 | β = 0.94 | 0.43-1.45 | <0.001 |

Notes. Models adjusted for age, sex, and diagnosis (MDD vs BD). Ordinal outcomes were analyzed using proportional-odds ordinal logistic regression (reported as OR). PHQ-9 and GAD-7 were analyzed using linear regression with z-standardized outcomes (reported as β). Reference group = Cluster 3 (Lower-severity).

**Supplementary Table S3. Age moderation and age-stratified sensitivity analyses**

In the stratified analyses (12-17 vs 18-21 years), the 12-17 group comprised n = 273 (Cluster 1 n = 106, Cluster 2 n = 73, Cluster 3 n = 94), whereas the 18-21 group comprised n = 38 (Cluster 1 n = 6, Cluster 2 n = 6, Cluster 3 n = 26).

**A) Cluster × Age interaction tests (12-21 years)**

| **Outcome** | **N** | **Test** | **F** | **df1** | **df2** | **p** |
| --- | --- | --- | --- | --- | --- | --- |
| Pain perception | 311 | Joint test of Cluster×Age terms | 0.909 | 2 | 304 | 0.404 |
| NSSI frequency (month) | 311 | Joint test of Cluster×Age terms | 1.397 | 2 | 304 | 0.249 |
| NSSI frequency (year) | 311 | Joint test of Cluster×Age terms | 0.572 | 2 | 304 | 0.565 |
| Age of first NSSI | 311 | Joint test of Cluster×Age terms | 1.938 | 2 | 304 | 0.146 |

**B) Age-stratified sensitivity analyses (12-17 vs 18-21 years)**

| **Outcome** | **Age stratum** | **N** | **Test** | **F** | **df1** | **df2** | **p** |
| --- | --- | --- | --- | --- | --- | --- | --- |
| Pain perception | 12-17 | 273 | Overall cluster effect (adjusted) | 1.738 | 2 | 268 | 0.178 |
| Pain perception | 18-21 | 38 | Overall cluster effect (adjusted) | 1.043 | 2 | 33 | 0.364 |
| NSSI frequency (month) | 12-17 | 273 | Overall cluster effect (adjusted) | 13.822 | 2 | 268 | 1.94e-06 |
| NSSI frequency (month) | 18-21 | 38 | Overall cluster effect (adjusted) | 0.025 | 2 | 33 | 0.976 |
| NSSI frequency (year) | 12-17 | 273 | Overall cluster effect (adjusted) | 9.714 | 2 | 268 | 8.46e-05 |
| NSSI frequency (year) | 18-21 | 38 | Overall cluster effect (adjusted) | 0.408 | 2 | 33 | 0.668 |
| Age of first NSSI | 12-17 | 273 | Overall cluster effect (adjusted) | 0.903 | 2 | 268 | 0.407 |
| Age of first NSSI | 18-21 | 38 | Overall cluster effect (adjusted) | 1.189 | 2 | 33 | 0.317 |

**Notes.** Interaction models were estimated as Outcome ~ C(cluster)×age + C(sex) (age treated as continuous). Age-stratified models were estimated within each stratum as Outcome ~ C(cluster) + age + C(sex). For these reviewer-requested sensitivity analyses, outcomes were treated as ordered numeric scores and tested using general linear models so that joint F tests of Cluster × Age terms and overall adjusted cluster effects could be reported consistently across outcomes.
